# Histone Modifications and Other Facets of Epigenetic Regulation in Trypanosomatids: Leaving Their Mark

**DOI:** 10.1128/mBio.01079-20

**Published:** 2020-09-01

**Authors:** Swati Saha

**Affiliations:** a Department of Microbiology, University of Delhi South Campus, New Delhi, India; University of Texas Health Science Center at Houston; University of Texas Health Science Center at Houston

**Keywords:** histone modifications, chromatin modifications, chromatin architecture, histone acetylation, histone acetyltransferases, histone methylation, histone methyltransferases, histone modification readers, *Leishmania*, *Trypanosoma*, trypanosomes, trypanosomatids, protozoan parasite, protozoan 3D genome architecture

## Abstract

Histone posttranslational modifications (PTMs) modulate several eukaryotic cellular processes, including transcription, replication, and repair. Vast arrays of modifications have been identified in conventional eukaryotes over the last 20 to 25 years. While initial studies uncovered these primarily on histone tails, multiple modifications were subsequently found on the central globular domains as well. Histones are evolutionarily conserved across eukaryotes, and a large number of their PTMs and the functional relevance of these PTMs are largely conserved. Trypanosomatids, however, are early diverging eukaryotes. Although possessing all four canonical histones as well as several variants, their sequences diverge from those of other eukaryotes, particularly in the tails. Consequently, the modifications they carry also vary. Initial analyses almost 15 years ago suggested that trypanosomatids possessed a smaller collection of histone modifications. However, exhaustive high resolution mass spectrometry analyses in the last few years have overturned this belief, and it is now evident that the “histone code” proposed by Allis and coworkers in the early years of this century is as complex in these organisms as in other eukaryotes. Trypanosomatids cause several diseases, and the members of this group of organisms have varied lifestyles, evolving diverse mechanisms to evade the host immune system, some of which have been found to be principally controlled by epigenetic mechanisms. This minireview aims to acquaint the reader with the impact of histone PTMs on trypanosomatid cellular processes, as well as other facets of trypanosomatid epigenetic regulation, including the influence of three-dimensional (3D) genome architecture, and discusses avenues for future investigations.

## Abstract (precis)

Histone posttranslational modifications (PTMs) modulate several eukaryotic cellular processes, including transcription, replication, and repair. Vast arrays of modifications have been identified in conventional eukaryotes over the last 20 to 25 years. While initial studies uncovered these primarily on histone tails, multiple modifications were subsequently found on the central globular domains as well. Histones are evolutionarily conserved across eukaryotes, and a large number of their PTMs and the functional relevance of these PTMs are largely conserved.

## INTRODUCTION

Trypanosomatids are digenetic protozoan parasites causing debilitating diseases that collectively affect tens of millions in tropical and subtropical countries. They primarily afflict the world’s poor who do not have access to clean housing and sanitation and whose immune systems are weak due to undernourishment/malnutrition. Among the most dangerous of the trypanosomatids is Trypanosoma brucei, causing human African trypanosomiasis (sleeping sickness). As initial symptoms are unspecific, the parasite remains undetected at first, multiplying extracellularly in the bloodstream and lymphatic vessels, eventually crossing the blood-brain barrier to cause sleep disorder, seizures, coma, and death. Trypanosoma cruzi causes Chagas disease. Endemic to Latin American countries, here too the initial acute phase of infection is frequently asymptomatic or accompanied by generic symptoms. The later chronic phase, however, is often coupled to serious digestive and cardiac disorders. The various *Leishmania* species cause leishmaniases, distributed over Central and South America, West and South Asia, Southern Europe, and Northern Africa. Leishmaniases manifest in three forms: cutaneous, subcutaneous, and visceral. Cutaneous leishmaniasis causes disfiguring skin lesions, subcutaneous leishmaniasis is characterized by a destruction of the mucocutaneous membranes of the nasal and oral cavities, and visceral leishmaniasis is typified by irregular fever spells, weight loss, enlarged liver and spleen, and anemia and can be fatal. The diseases caused by trypanosomatids are curable if treated in a timely manner, but due to the toxic side effects of existing chemotherapeutic treatments as well as emerging drug resistance, the hunt for new drug targets continues. Investigations into the cellular biology of these parasites entered a new era with the unveiling of their genome sequences (1–3), combined with the advent of new high-throughput technologies. Studies of epigenetic control circuits have yielded significant information on mechanisms regulating various processes in these organisms. This minireview focuses on highlighting recent advances in our knowledge of the impact of histone modifications and other aspects of chromatin organization in these parasites.

## ORGANIZATION OF CHROMATIN AND IMPORTANCE OF HISTONE MODIFICATIONS

The organization of eukaryotic DNA into chromatin renders it compact enough to fit into the nucleus, simultaneously protecting it from damage. The ∼3.3 Gb human genome fits into a nucleus with a diameter of merely 10 to 15 μm, while the ∼35-Mb trypanosomatid genomes are accommodated in an ∼2 μm-diameter nucleus. The basic unit of chromatin is the nucleosome, comprising a core around which the DNA is wrapped (4, 5). The octameric core consists of four highly basic histone proteins: H2A, H2B, H3, and H4. H3 and H4 form a tetramer (H3_2_-H4_2_) that defines the diameter of the nucleosomal particle, while H2A-H2B form a heterodimer, with two heterodimers in a nucleosome. The histones comprise a central globular domain or “histone fold,” from which flexible N- and C-terminal “tails” extend. While the length of DNA in a nucleosome varies between ∼150 and 260 bp, the DNA that is directly wrapped around the core is fixed at 145 to 147 bp (core DNA), with the rest forming the linker DNA between adjacent nucleosomes. The linker DNA is partly coated with another histone of similar domain structure, H1. The array of nucleosomes so formed compresses the genome ∼6-fold into fibers of 10 nm diameter. These form higher-order structures by interactions between the nucleosomes involving their tails. Histone H1 promotes the formation of these higher-order structures. The trypanosomatid core histones are structurally conserved with those of other eukaryotes. However, H1 deviates, with no central globular domain and a rather long C-terminal tail. Consistent with this, *in vitro* studies demonstrated that chromatin does not form highly condensed structures in these organisms (6). The Saccharomyces cerevisiae H1 is also deviant from those of filamentous fungi and higher eukaryotes, as it has two globular domains, while Schizosaccharomyces pombe is devoid of H1. This is in keeping with the fact that trypanosomatids as well as the two yeasts undergo a closed mitosis, and thus, their chromatin does not form the highly condensed structures seen in higher eukaryotes.

Histones carry several posttranslational modifications (PTMs). The earliest data identifying histone acetylation and methylation (in calf thymus nuclei) were reported by Allfrey et al. (7), who proposed reversible histone acetylation to regulate activation and repression of RNA synthesis. Thirty years later, the first histone acetyltransferase was identified by the Allis laboratory, who purified an enzyme from *Tetrahymena* that could acetylate calf thymus histones (8). The next 2 decades witnessed an explosion of discoveries. Several histone modifications were identified, many of them conserved. It became apparent early on that the global histone modification landscape is dynamic, being governed by the cellular milieu and external cues. Furthermore, the distribution of the various histone modifications at any given time is not uniform across the genome. Histone modifications modulate chromatin-linked processes through two broad mechanisms: one, by causing chromatin to adopt a more open/compact conformation, and two, by marking specific residues which could serve as landing pads for proteins that possess recognition modules for those modification marks. Strahl and Allis proposed that the modifications on the histone tails form a “histone code” that is “read” by various effector proteins of downstream processes (9). Over 20 different modification types have now been identified. These include acetylation, methylation, phosphorylation, ubiquitination, sumoylation, proline isomerization, propionylation, butyrylation, citrullination, among others (10, 11). Three groups of proteins modulate histone modifications and their impact: the “writers,” “erasers,” and “readers.” Over 50 “writers” and about twice as many “readers” have been identified thus far. The trypanosomatid proteins of these different categories will be discussed below.

## LANDSCAPE OF HISTONE MODIFICATIONS IN TRYPANOSOMATIDS

The early diverging trypanosomatids possess all four canonical core histones as well as four variant histones, H2A.Z, H2.V, H3.V, and H4.V, although H4.V has been identified only in T. brucei so far. As their histones are divergent from those of conventional eukaryotes (they share 40 to 60% identity with the core histones of higher eukaryotes S. cerevisiae, S. pombe, Drosophila melanogaster, Caenorhabditis elegans, Mus musculus, and Homo sapiens), as well as those of early diverging eukaryotes of other groups (sharing 35 to 60% identity with the core histones of Plasmodium falciparum, Toxoplasma gondii, Cryptosporidium parvum, and Trichomonas vaginalis), the repertoire of PTMs they carry also vary. This is in contrast to apicomplexans *Plasmodium* and *Toxoplasma* whose core histone sequences share 65 to 95% identity with those of the conventional eukaryotes listed above. The first investigations into global histone modifications in T. brucei using a combination of Edman degradation and mass spectrometry (12, 13) identified several acetylation and methylation marks on the N-terminal tail of H4, but fewer distinct modifications in H3. In a conspicuous variation from other eukaryotes, the C-terminal tail of H2A was hyperacetylated. Mass spectrometry analysis also identified distinct acetylated and methylated lysine residues in T. cruzi H4 (14). The identification of relatively few modification marks led to the conclusion that trypanosomatids have a simple “histone code.” Two comprehensive investigations using high-resolution mass spectrometry carried out a decade later demonstrated otherwise (15, 16). Over ∼170 PTMs were identified in the tails and globular domains of the canonical and variant histones of T. cruzi. These included 13 distinct types of PTMs, eight of which were detected in trypanosomatids for the first time. While the tails were enriched in acetylation marks, the alternative acylation modifications were largely confined to the globular domains. In addition to H2A hyperacetylation at the C-terminal tail, the variant H2A.Z was hyperacetylated on the N- and C-terminal tails and hyperphosphorylated on the N-terminal tail. H2B had several acetyllysine and methyllysine residues as well as alternative acylation modifications in its globular domain, while H2B.V was hyperacetylated on the N-terminal tail. H3 was mostly acetylated at its N terminus and methylated in its globular domain, and H4 carried a large number of acetylation and methylation marks at the N-terminal tail as well as within the globular domain. A recent study (published while this article was under review) has identified the histone PTMs associated with the distinctive T. cruzi parasite life forms, with over 60 PTM fluctuations during the life cycle (17). Two independent studies (one of which was published while this article was under review) have recently unearthed more than 150 PTMs on T. brucei histones also (18, 19). Strikingly, virtually no acetylation marks were detectable in H3.V and H4.V. Kraus et al. (18) compared the histone acetylation and methylation marks on nucleosomes at transcription start sites (TSSs) with those on nucleosomes at other sites in T. brucei and found some modifications to be specific to TSSs while others were universally distributed. Histone modifications have been globally identified in *Plasmodium* and *Toxoplasma* as well (20, 21). Over 230 modifications have been identified in the intraerythrocytic stages of *Plasmodium*, an organism with a complex life cycle. Several modifications found in *Plasmodium* and *Toxoplasma* histones are conserved with those found in humans, although many are unique to these organisms (21).

The global landscape of histone modifications has not been reported in *Leishmania* species. As histones are largely conserved between *Leishmania* and *Trypanosoma* species, it could be conjectured that the PTMs and their functional roles will also be largely conserved. However, this may not necessarily be so, as the three parasites have different lifestyles. While T. brucei exists and multiplies as an independent form in the bloodstream of the mammalian host, T. cruzi propagates in the cytosol of mammalian host cells, and *Leishmania* species survive and propagate in the phagosomes of host macrophages. Indeed, though several identified histone PTMs were conserved between T. brucei and T. cruzi, others were unique to one or the other. Leishmania donovani H4 has been identified to be acetylated at K4 and K10 (22, 23), and Leishmania major H3 has been reported to be acetylated at its N-terminal tail (24). H3K4 (histone H3 lysine 4) is conserved in *Leishmania* species, and work carried out in our laboratory indicates that it is trimethylated in L. donovani promastigotes (insect form; unpublished results).

Some trypanosomatid histone modifications may be synonymous with modifications in histones of model eukaryotes. Thus, the trypanosomatid H3methylK76 mark may match the H3methylK79 mark seen in other eukaryotes, the H4acetylK4 mark may correspond to the universal H4acetylK5 modification, the H4methylK17/18 marks may match the conserved H4methylK20, and H3K23 and H3K32 may be synonymous with the universal H3K27 and H3K36 residues. Indeed, functional similarity was evident in the case of the H3K76me/H3K79me marks of T. brucei and mammalian cells, respectively, with both playing a role in DNA replication (25, 26).

## WRITERS AND ERASERS

**FIG 1:**
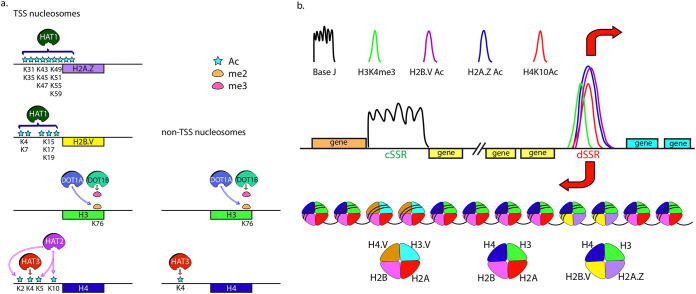
(a) The enzymes that have been identified to modify histones at specific sites. The target substrates of the specific enzymes are indicated for each enzyme. The modifications on TSS nucleosomes versus non-TSS nucleosomes come from the work of Kraus et al. (18). (b) The epigenetic landscape associated with PolII-mediated transcriptional events. Arrows indicate the direction of transcription. The divergent strand switch regions (dSSRs) are enriched in H3K4me3, H4K10Ac, acetyl-H2A.Z, and acetyl-H2B.V. The convergent strand switch regions (cSSRs) are enriched in base J, H3.V, and H4.V.

The “writers” of histone modifications enzymatically add modifications on amino acids, while the “erasers” remove the modifications. The identification of relatively few “writers” and “erasers” in initial annotations of the trypanosomatid genome sequences suggested lack of functional redundancy and easier decoding of the roles of individual histone modifications. Subsequently, however, a larger number of such proteins were identified (Table 1). Only a few have been characterized. The “writers” whose histone target sites have been identified are portrayed in Fig. 1a.

**TABLE 1:** The writers and erasers of trypanosomatid histone modifications

Class of modifier	Type	Organism	No. of modifiers identified through genome sequence	Protein	Histone substrate if identified	Functional role(s) if known (reference)^*a*^
Histone acetyltransferase	MYST family	T. brucei	3	HAT1	H2A.Z	Essential. Nuclear. Replication, transcription (35, 18).
				HAT2	H4K10	Essential. Nuclear. Transcription (35, 18).
				HAT3	H4K4	Nonessential. Nuclear. DNA repair (36, 89).
		T. cruzi	4	HAT1		
				HAT2		
				HAT3		
				HAT4		
		*Leishmania* species	4	HAT1		
				HAT2	H4K10	Essential. Nuclear. Transcription (19).
				HAT3	H4K4	Nonessential. Nuclear. DNA repair (20).
				HAT4	H4K14 *in vitro*	Nonessential. Cytosolic except at G_2_M. Cell cycle control (37, 38).
	GNAT family	T. brucei	2	Elp3a		Nonessential. Nuclear. No role determined yet (39).
				Elp3b		Nonessential. Nuclear. rDNA transcription (39).
		T. cruzi	2	Elp3a		
				Elp3b		
		*Leishmania* species	2	Elp3a		
				Elp3b		
Histone deacetylase	Zn^2+^- dependent HDACs	T. brucei	4	DAC1		Essential. Nuclear. Telomeric silencing antagonist (24, 25).
				DAC2		Nonessential. Cytoplasmic (24).
				DAC3		Essential. Nuclear. Silencing of ESs of VSGs (24, 25).
				DAC4		Nonessential. Cytoplasmic. Cell cycle progression (24).
		T. cruzi	4	DAC1		
				DAC2		
				DAC3		
				DAC4		
		*Leishmania* species	3	DAC1		
				DAC3		
				DAC4		
	NAD^+^- dependent sirtuins	T. brucei	3	Sir2rp1	H2A, H2B *in vitro*	Nonessential. Nuclear. DNA repair. Subtelomeric transcription control (26, 27, 28).
				Sir2rp2		Nonessential. Mitochondrial.
				Sir2rp3		Nonessential. Mitochondrial.
		T. cruzi	2	Sir2rp1		Cytoplasmic.
				Sir2rp3		Mitochondrial.
		*Leishmania* species	3	Sir2rp1		Essential. Cytoplasmic. α-Tubulin deacetylation.
				Sir2rp2		Mitochondrial.
				Sir2rp3		Mitochondrial.
Histone methyltransferase	SET domain-containing	T. brucei	∼25–30			
		T. cruzi	∼25–30			
		*Leishmania* species	∼25–30			
	DOT1- related	T. brucei	3	DOT1A	H3K76me2	Essential. Replication (22, 41).
				DOT1B	H3K76me3	Nonessential. VSG silencing and transcriptional switching, cell cycle control (41, 65).
				DOT1 putative		
		T. cruzi	3	DOT1A		
				DOT1B	H3K76me3	Nonessential. Promotes mitosis and cytokinesis (43).
				DOT1 putative		
		*Leishmania* species	3	DOT1A		
				DOT1B		
				DOT1 putative		
	PRMT	T. brucei	5	PRMT1		Nonessential. Regulation of cell’s response to starvation.
				PRMT3		
				PRMT5		Nonessential. Cytoplasmic.
				PRMT6	H3 and H4 *in vitro*	Nonessential. Regulation of cytokinesis. Interacts with core histones (46).
				PRMT7	H4 peptides *in vitro*	Nonessential. Cytoplasmic (45).
		T. cruzi	5	PRMT1		
				PRMT3		
				PRMT5		
				PRMT6		
				PRMT7		
		*Leishmania* species	5	PRMT1		
				PRMT3		
				PRMT5		
				PRMT6		
				PRMT7		Nonessential. Cytosolic. RNA biology (45).
Histone demethylases	Jumonji family	T. brucei	4			
		T. cruzi	4			
		*Leishmania* species	4			

a References are cited here only where related to histones and/or DNA-related processes.

### Histone deacetylases.

The “erasing” of acetyl groups from acetyllysine residues restores the positive charge on lysines, tightening histone-DNA contacts. When the four Zn^2+^-dependent T. brucei histone deacetylases (HDACs) were investigated, only DAC1 and DAC3 were found to be essential (27). Both were nuclear (28). The bloodstream T. brucei is sheathed with glycoprotein, and a large collection of genes (variant surface glycoprotein [VSG] genes) encode these surface glycoprotein molecules. The organization of the VSG genes was primarily uncovered from two major genome sequencing studies of T. brucei (1, 29), one of which employed single-molecule real-time (long-read) sequencing methodology (29). The data collectively revealed that the vast majority of the repertoire of ∼2,500 VSG genes lie in long subtelomeric arrays on the diploid genome. Several of these are pseudogenes, and transcriptome sequencing (RNA-seq) analysis has confirmed that none of the genes in these arrays are expressed (29, 30). Some VSG genes lie within expression sites (ESs) located near telomeres, as part of polycistronic units that are transcriptionally competent. There are 15 such ESs in bloodstream T. brucei, and at a given time, only one VSG gene lying in an ES is expressed. The parasite evades the host immune system by periodically switching the VSG being expressed. VSG switching occurs either through recombinational events triggered by DNA damage at the VSG loci within the ESs or through transcriptional switching. Recombination-based VSG switching at the active ESs initiates due to breaks that occur during the natural course of events in these regions of relative genomic instability. The break leads to DNA resection, the production of single-stranded DNA, and homology-based DNA repair. The subtelomeric arrays of inactive VSG genes contribute to the repertoire of sequences that are drawn upon for this process, and thus, an active VSG gene can be replaced by another one, leading to a change in the surface coat protein (30). The recombination-based switch, as well as the transcriptional switch that shuts off the expression of a VSG in one ES and turns on expression of another VSG in a different ES, is regulated mainly by epigenetic mechanisms. Wang et al. (28) found that while DAC3 was essential for silencing VSG expression site promoters, DAC1 had an “antagonistic effect” on telomeric silencing. Although both DACs exhibited histone deacetylase activity *in vitro*, to date, the *in vivo* substrates of these HDACs have not been identified. HDACs have not been characterized in the other trypanosomatids, and a DAC2 ortholog is absent in *Leishmania* species.

Sirtuins are Sir2-related NAD^+^-dependent deacetylases. Of the three sirtuins identified in trypanosomatids (Sir2rp1 to Sir2rp3), Sir2rp2 is absent in T. cruzi. TbSir2rp1 was found to be a nuclear protein that deacetylated as well as ADP-ribosylated histones H2A and H2B *in vitro* (31, 32), but none of the histones could be identified as an *in vivo* substrate. TbSir2rp1 repressed subtelomeric transcription without affecting antigenic variation, in addition to playing a role in DNA repair (31, 33). None of the T. cruzi and *Leishmania* sirtuins have been found to act on acetyl-histones *in vitro* or *in vivo*, nor have they been linked to any DNA-related process (34, 35). This is not unusual, as the role of sirtuins is now known to extend beyond the targeting of histone substrates, deacetylating proteins not only in the nucleus but also in the cytoplasm and mitochondrion.

### Histone acetyltransferases.

Histone acetyltransferases (HATs) “write” acetyl marks on lysine residues, neutralizing their positive charge by catalyzing the transfer of an acetyl group from acetyl coenzyme A (acetyl-CoA). This loosens histone-DNA contacts, promoting a more accessible conformation of chromatin. The acetyllysine modification may also serve as a recruitment site for “readers” carrying bromodomains (discussed later). Broadly categorized as type A and type B, type A HATs are typically nuclear and acetylate nucleosomal histones, while type B HATs are cytosolic and acetylate freshly synthesized free histones. Classified into MYST, GNAT, and p300/CBP families, type A HATs regulate transcriptional activation, origin activation, replication timing, and DNA repair. The HATs are the most extensively characterized trypanosomatid “writers.” While T. cruzi and *Leishmania* species harbor four MYST family HATs, T. brucei has only three. All possess two GNAT family HATs.

MYST (named after MOZ, Ybf2/Sas3, Sas2, and Tip60) family members regulate DNA replication, transcription, and DNA repair (36–38) and are often an inherent component of a much larger complex (example, Esa1 is a part of the yeast NuA4 complex [39]). The three T. brucei MYST family HATs 1 to 3 were all found to be nuclear, and only T. brucei HAT1 (TbHAT1) and TbHAT2 were essential. The three HATs have distinct substrates (Fig. 1a)—TbHAT1 acetylates multiple residues at the N termini of H2A.Z and H2B.V, TbHAT2 primarily acetylates H4K10, and TbHAT3 acetylates H4K4 (18, 40, 41). Of the L. donovani MYST family HATs, L. donovani HAT2 (LdHAT2) and LdHAT3 were found to be nuclear, while LdHAT4 was cytosolic except at G_2_/M when it was also in the nucleus. These HATs also had distinct substrates, with LdHAT2, LdHAT3, and LdHAT4 targeting H4K10, H4K4, and H4K14, respectively, though H4K14 could not be confirmed as the *in vivo* substrate of LdHAT4 due to nonavailability of modification-specific antibodies. Only LdHAT2 was essential for normal growth, though absence of LdHAT4 was coupled to G_2_/M defects (22, 23, 42, 43). The distinctive nature of the target sites of these HATs suggests the absence of functional redundancy among them, quite unlike other eukaryotes where the same HAT has multiple substrates: for example, the yeast Esa1 acetylates four H4 residues and also acetylates H2A.Z. The roles of the trypanosomatid MYST family HATs and the histone acetylations they mediate will be discussed later.

The two trypanosomatid GNAT family HATs are Elp3 orthologs. Elp3 is a component of the multisubunit Elongator complex that facilitates transcriptional elongation. Trypanosomatids are the only eukaryotes where two Elp3 orthologs (Elp3a and Elp3b) have been identified. Examined so far in T. brucei only, both are nuclear, though their subnuclear distribution patterns are dissimilar (44). T. brucei RNA polymerase I (PolI) transcribes the ribosomal DNA (rDNA) locus as well as the VSG gene being expressed at the time. Alsford and Horn (44) found that TbElp3b negatively modulated rDNA transcription, without having any effect on monoallelic VSG transcription. Target histone substrates for the TbElp3 proteins have not been identified thus far.

### Histone methyltransferases.

Histone methyltransferases catalyze the methylation of lysine or arginine residues using *S*-adenosylmethionine (SAM) as the methyl group donor. The lysine methyltransferases mono-, di-, and/or trimethylate lysines and are either SET [su(var)3-9, enhancer of zeste, trithorax] domain proteins, or DOT1 (disruptor-of-telomeric silencing)-related proteins. The protein arginine methyltransferases (PRMTs) either monomethylate or dimethylate arginines. Histone methylation events can activate or repress transcription based on the site of methylation and the genomic context. The methylated residues serve as anchor sites for various “readers” (discussed later).

Early annotations of trypanosomatid genome sequences uncovered the presence of two DOT1 and three SET domain histone methyltransferases (45) in addition to five PRMTs. A third DOT1 ortholog and between 25 and 30 SET domain proteins were later identified. The presence of multiple DOT1 proteins is quite unlike yeast, *Drosophila*, and humans who have only one DOT1 ortholog. None of the putative SET domain methyltransferases have been characterized, and the histone target site of only two methyltransferases—DOT1A and DOT1B—have been determined (Fig. 1a). Using DOT1B knockout and DOT1A knockdown lines in combination with mass spectrometry analysis, coupled with analysis using modification-specific antibodies, Janzen et al. (46) ascertained that the essential DOT1A was responsible for dimethylation of H3K76 and the nonessential DOT1B mediated trimethylation of the same residue. The DOT1 orthologs displayed a temporally regulated mode of action with DOT1A catalytic action preceding DOT1B action (47). DOT1B has recently been shown to mediate trimethylation of H3K76 in T. cruzi as well (48). Expression of the TbDOT1 orthologs in a *dot1Δ* budding yeast strain, followed by mass spectrometry analysis, revealed that the TbDOT1 proteins methylated H3K79 in yeast, strongly suggesting that the trypanosomatid H3K76 methylation mark was synonymous with the H3K79 methylation mark of conventional eukaryotes (49).

Although the T. brucei PRMTs and *Leishmania* PRMT7 have been examined and TbPRMT7 has been found to methylate H4-derived peptides *in vitro* while TbPRMT6 methylates both H3 and H4 *in vitro*, there is no evidence of *in vivo* histone methylation by any of these PRMTs (50–54).

## ROLE OF HISTONE MODIFICATIONS IN TRYPANOSOMATID NUCLEOSOMAL DEPOSITION

Early studies with *Tetrahymena*, *Drosophila*, and human cells revealed that while newly synthesized histones were diacetylated at H4K5,K12, steady-state histones were not—leading to the conclusion that H4K5,K12 diacetylation is needed for establishment but not maintenance of chromatin structure (55). As H4 was acetylated at K12 by a type B (cytosolic) *Drosophila* HAT *in vitro* (56), it was believed that type B HATs mediate histone H4 diacetylation in the cytosol prior to nuclear import and nucleosomal deposition. Subsequent studies in yeast revealed that transcriptional start sites were enriched in H4 acetylation at K5, K8, K12, and K16, suggesting that though perhaps not required for maintenance of chromatin structure, they played a role beyond histone deposition (57).

The role of histone modifications in trypanosomatid nucleosomal deposition has not been investigated in depth. However, studies in L. donovani have shown that acetylation at H4K4 is critical for histone deposition—H4 devoid of K4 acetylation remained mostly in the soluble fraction of cell extracts. Further, no H4acetylK4 was detectable in the cytosol in immunofluorescence analysis, suggesting that this acetylation was occurring in the nucleus prior to deposition (23). Treatment of T. brucei cells with cycloheximide resulted in the complete absence of unmodified H4K4, indicating that this residue was acetylated very rapidly after histone synthesis and was not actively deacetylated by histone deacetylases, in accordance with the fact that >70% of T. brucei H4K4 sites are acetylated (41). Thus, it appears that while the trypanosomatid H4K4 acetylation event may be synonymous with the H4K5 acetylation event of conventional eukaryotes in that it precedes histone deposition, it differs from it as it persists on assembled chromatin. The deficiencies in nucleosomal assembly linked to downregulation of H4K4 acetylation in the absence of LdHAT3 is the most likely cause of partial viability defects in these cells (23). However, neither LdHAT3 nor TbHAT3 were absolutely essential during normal growth, possibly because of residual H4K4 acetylation (mediated by an alternative HAT), as detected in LdHAT3-null cells.

## EPIGENETIC PROCESSES MODULATING TRYPANOSOMATID GENE EXPRESSION

The uniqueness of trypanosomatid genomes is reflected in their unusual organization. Tens to hundreds of functionally unrelated genes are unidirectionally clustered, with adjacent clusters usually lying on opposite strands of the genome. The strand switch region (SSR) between two adjacent clusters is either divergent (dSSR) or convergent (cSSR) depending on whether the clusters are directed away from or toward each other, respectively. Occasional head-to-tail clusters are also seen. Nuclear run-on assays with asynchronously growing L. major have revealed that the genes of a cluster are coordinately transcribed, with transcription initiating bidirectionally from the dSSRs and terminating in the cSSRs (Fig. 1b). This polycistronic transcription is followed by trans-splicing of a short leader sequence and a poly(A) tail onto the 5′ and 3′ ends of the individual protein-coding units (58–60). No consensus sequences have been identified at the dSSRs, although a poly(C) tract at the region where transcription initiates in the chromosome 1 dSSR is conserved across 15 *Leishmania* species (61). In the absence of defined regulatory sequence elements at the transcription start sites (TSSs) as well as the apparent absence of canonical transcription factors that are generally conserved across eukaryotes, epigenetic control mechanisms may play a major role in modulating gene expression.

Studies of conventional eukaryotes have found histone variants H2A.Z and H3.3 to form less stable nucleosomes relative to the canonical H2A and H3 (62). The enrichment of promoters of protein-coding genes with these variants suggests they facilitate the unraveling of nucleosomal structure during transcriptional activation. A genome-wide analysis of the distribution of the T. brucei H2A.Z, H2B.V, H3.V, and H4.V using Chip-Seq (chromatin immunoprecipitation with DNA sequencing) revealed TSSs to be enriched in H2A.Z and H2B.V and transcription termination sites to be enriched in H3.V and H4.V (63). Nucleosomes containing H2A.Z and H2B.V were relatively unstable, and taken collectively, the data suggest that the presence of nucleosome-destabilizing variant histones at transcription start sites is an evolutionarily conserved mechanism linked to transcriptional activation. The distribution of histone variants in *Plasmodium* species is somewhat different, with H2A.Z and H2B.Z being positioned in nucleosomes at all intergenic regions of the genome, perhaps linked to the very high AT content (64). The variants H3.V and H4.V were found to control VSG gene silencing at T. brucei ESs when results of ATAC-Seq (assay for transposase-accessible chromatin using sequencing) studies established that VSG genes at ESs in T. brucei cells devoid of H3.V and H4.V were more accessible than in wild-type cells, signifying a more open conformation. This was linked to increased frequency of recombination-based VSG switching as well as partial derepression of VSG gene silencing at these sites, sometimes resulting in the simultaneous expression of more than one VSG (29). These data add a new dimension to our knowledge of how antigenic variation is controlled epigenetically in this seemingly simple, yet so artful pathogen.

Acetylation at H3K9/K14 and H4K5/K12 and trimethylation at H3K4 are linked to transcriptional upregulation across eukaryotes and have been observed in *Plasmodium* and *Toxoplasma* also, although most of the *Plasmodium* euchromatic genome (not only active promoter regions as in higher eukaryotes) carries H3K4me3 and H3K9Ac marks—in active promoters and 5′ ends of genes, as well as in intergenic regions (65–67). One unusual feature of trypanosomatids is that though they harbor the ubiquitous H3K4 residue, the universal H3K9 residue, whose methylation is critical to gene silencing in other eukaryotes, is absent. It is unlikely that the H3K10 residue is synonymous with the conventional H3K9 residue, as H3K10me3 (in addition to H3K14Ac, H3K23Ac, and H3K27Ac) is highly enriched at the active L. major rDNA promoter region (68). However, these data were obtained using commercially available antibodies designed for conventional eukaryotic histones whose sequences flanking the pertinent residue are highly divergent from those of the trypanosomatid H3, underscoring the importance of generating modification-specific antibodies to trypanosomatid histone sequences for optimal analysis. Histone modification marks at TSSs were first examined in T. cruzi by Respuela et al. (69) who observed the dSSRs to be enriched in acetyl-H3 and acetyl-H4 in addition to H3trimethylK4. Thereafter, the T. brucei dSSRs were determined to be enriched in H4K10 acetylation and H3K4 trimethylation. The two peaks overlapped, with H3K4me3 peaks skewed slightly toward the upstream of the H4K10Ac peaks (63, 70). T. brucei mononucleosomes carrying H2B.V were enriched in H3K4me3 compared to those carrying the canonical H2B, implicating H2B.V in transcriptional activation (71). Around the same time, L. major dSSRs/TSSs were found to be to be enriched in H3 acetylation (24), and later studies revealed L. donovani TSSs to be enriched in H4acetylK10 as well (22). The overexpression of H4K10R and H4K14R in T. cruzi resulted in growth defects and aberrant patterns of transcription and replication (72), implicating a universal role for H4K10 acetylation in transcriptional events in this group of organisms. Recent mass spectrometry analyses identified enrichment of additional acetylation and methylation marks on nucleosomes at T. brucei TSSs. These included H2A.Z and H2B.V hyperacetylation and H3 methylations. The well characterized H3K76 methylation marks and the H4K4Ac modification were equally detected at TSS and non-TSS nucleosomes, ruling out a role for these two modifications in PolII-mediated transcription (18). Nevertheless, the DOT1B protein that mediates H3K76 trimethylation tightly regulates the silencing and transcriptional switching of PolI-transcribed VSGs, with deletion of DOT1B resulting in the occasional simultaneous expression of two VSGs (73). The landscape of epigenetic marks associated with PolII-mediated transcriptional events is summarized in Fig. 1b.

The most conserved of the variant histones, H2A.Z has been found at active as well as inactive promoters in other eukaryotes, but acetylated H2A.Z has been detected only at active promoters (74, 75). In yeast, the acetylation of H4 tails promotes deposition of H2A.Z into nucleosomes. In human cells, H2A.Z localizes upstream and downstream of TSSs (with H3K4me3 localizing to TSSs). As the same HAT acetylates H4 and H2A.Z (Esa1 in yeast and Tip60 in human cells), the *in vivo* role of H2A.Z acetylation has not been confirmed in conventional eukaryotes, though it is hypothesized that H2A.Z acetylation regulates transcription by having a destabilizing effect on nucleosomal structure (62). With the acetylation of H4 and H2A.Z being mediated by different HATs in T. brucei, it was possible to dissect the significance of the two sets of acetylation events (18). Depletion of TbHAT1 was coupled to downregulation of H2A.Z and H2B.V acetylation without any impact on nucleosomal deposition of the two variants. This was accompanied by decreased transcript levels, signifying a role for H2A.Z hyperacetylation in setting up the chromatin environment for effective transcriptional activation. Interestingly, TbHAT2 depletion not only led to substantial decrease of H4K10 acetylation and a concomitant loss of H2A.Z deposition at the TSSs, but it also resulted in an upstream shift of the TSS without impacting the actual level of transcription. The primary role of H4K10 acetylation in transcription thus appears to be the facilitation of H2A.Z deposition at TSSs. In the absence of H2A.Z, the transcriptional machinery may find an alternate start site in a more conducive location on the template (18). In synchrony with these findings, LdHAT2 depletion also did not have a global impact on transcript levels. However, a small group of genes were sensitive to H4K10 acetylation levels (22). The downregulation of these genes (in response to LdHAT2 depletion) expressed from promoters lying within the polycistronic transcription units (PTUs), may be linked to a different local chromatin environment. An in-depth comparative analysis of the nucleosomal composition as well as modifications at these TSSs versus the TSSs at dSSRs would be illuminating. In addition to its role in PolII-mediated transcriptional activation, TbHAT1 depletion abrogated telomeric silencing (40). As TbSir2rp1 was critical for basal telomeric silencing while DAC1 had an antagonistic effect on the same, Wang et al. (28) proposed that TbHAT1 and TbDAC1 may act synergistically to modulate Sir2rp1-mediated silencing through the regulation of histone acetylation levels that define the boundaries of silencing. H1 was also found to impact PolI-mediated transcription, repressing transcription at the silent VSG expression sites and modulating VSG switching (76, 77).

Epigenetic effects on gene expression in trypanosomatids are not restricted to the effects of histone variants, histone PTMs, and chromatin remodellers. *Trypanosoma* and *Leishmania* PolII transcription termination sites are enriched in an unusual thymine analog, β-d-glucosylhydroxymethyluracil (base J). Inhibition of base J synthesis led to genome-wide *Leishmania* transcription termination defects and consequent poor cell viability, and the absence of base J in T. brucei was accompanied by transcriptional read through as well as antisense transcription at cSSRs (78–80). The recent identification of Leishmania tarentolae base J-binding protein (JBP3) has expanded the possibilities in examining transcription termination mechanisms in these organisms (81).

## REGULATION OF DNA REPLICATION AND CELL CYCLE PROGRESSION BY HISTONE MODIFICATIONS

The modulation of DNA replication by chromatin environment was demonstrated almost 2 decades ago by the Grunstein laboratory, who established a role for histone acetylation in controlling the replication timing of S. cerevisiae origins by deleting the gene encoding the Rpd3 histone deacetylase which led to earlier firing of origins (82). More recently, H3K9 acetylation by Rtt109 has been detected at regions about to be replicated in S. cerevisiae, though the role of this modification remains undetermined (83). Histone methylation also regulates DNA replication: Yu et al. (84) discovered that G9a-mediated H3K56 monomethylation was responsible for anchoring PCNA to chromatin in G_1_, prior to DNA synthesis in S phase, with G9a disruption leading to replication defects.

Histone acetylation is implicated in origin activation in T. brucei and L. major, although no direct evidence has linked acetylation status with origin firing. Several initiator Orc1-binding sites identified on the T. brucei genome were found to lie very closely upstream to the H4K10 acetylation peaks previously identified by Siegel et al. (63), and of the ∼100 active origins identified by marker frequency analysis, about 40 lay in regions of H4K10 acetylation (85). Marques et al. (86) found the L. major origins to be wide zones, and in several (but not all) cases, the zones mapped to regions of H3 acetylation enrichment previously determined by Thomas et al. (24). Kawahara et al. (40) found that TbHAT1 depletion resulted in the cells entering mitosis without undergoing nuclear DNA replication. Considering this with the fact that several trypanosomatid origins overlap with transcription start regions that are enriched in acetyl-H2A.Z, one might speculate that H2A.Z acetylation is critical for origin activation.

The role of histone methylation in trypanosomatid DNA replication and cell cycle progression was unearthed by Janzen et al. who observed that loss of H3K76 methylation caused cell cycle defects in T. brucei (46). Further investigations revealed that DOT1A depletion resulted in cells undergoing nuclear division without the completion of DNA synthesis, while overexpression of DOT1A was coupled to continuous DNA synthesis, resulting in aneuploidy (25). A recent report suggests that DOT1B-mediated trimethylation of H3K76 allows T. cruzi cells to traverse mitosis and undergo cytokinesis: in the absence of DOT1B, H3K76 remained dimethylated, which somehow signaled a postmitotic arrest (48). Phosphorylation of T. cruzi H1 plays a role in cell cycle progression as well. H1 phosphorylation at mitosis is believed to promote chromosome condensation, presumably by loosening of H1-DNA contacts leading to easier access to chromosome condensation protein machinery (87).

## HISTONE MODIFICATIONS THAT MODULATE DNA REPAIR EVENTS

The classical example of a histone modification that regulates DNA repair events in conventional eukaryotes is the phosphorylation of the H2AX variant at Ser139 (referred to as γH2AX). This trademark of eukaryotic DNA damage signaling serves as a platform for the recruitment of DNA damage response machinery. The corresponding DNA damage-induced phosphorylation event in T. brucei was identified to be at Thr130 in H2A, based on the fact that phospho-H2A foci colocalized with damage-induced RAD51 foci (referred to as γH2A [88]). The γH2A modification is conserved in *Leishmania* species (Thr128). This modification has been used as a DNA damage marker in *Trypanosoma* as well as *Leishmania* studies (89–94).

H2A and H4 acetylation as well as H4K20 dimethylation have been implicated in the modulation of the mammalian cell’s response to DNA damage (95). Nardelli et al. (96) found that challenging T. cruzi cells with gamma irradiation led to hyperacetylation of H4K10 and H4K14 but decreased H4K4 acetylation. In contrast, exposing L. donovani cells to UV irradiation resulted in hyperacetylation of H4K4 (23). A role beyond histone acetylation was discovered when it was observed that LdHAT3 mediated the acetylation of PCNA (the DNA clamp protein that anchors replicative and repair polymerases to template DNA) *in vivo* in response to UV irradiation. HAT3-mediated PCNA acetylation was essential for the subsequent monoubiquitination of PCNA, a step that is important for facilitating the interaction of PCNA with translesion DNA polymerases (23). In the absence of LdHAT3, cells were unable to recover from the effects of UV irradiation. T. brucei HAT3 was also found to support DNA repair, when Glover and Horn (97) discovered that TbHAT3 and TbSir2rp1 impact DNA double-strand break repair in contrasting ways. While TbHAT3 promoted DNA resection, leading to single-stranded DNA (ssDNA) formation and RAD51 accumulation at internal chromosome break sites, Sir2rp1 promoted the dissociation of RAD51 filaments at these sites. TbHAT3 exerted an opposite effect at subtelomeric sites, thus suppressing recombination at VSG sites where Sir2rp1 had no significant role. The linker histone was also found to regulate repair events—the presence of H1 was found to generally hamper DNA repair at methyl methanesulfonate (MMS)-induced DNA damage sites (76).

## READERS AND CROSS TALK

Proteins that mediate DNA-related transactions often contain modules that recognize specific histone PTMs, thus facilitating their recruitment to the site of necessary action. A number of such “readers” have been identified during trypanosomatid genome sequence annotations, and some have been studied (Table 2).

**TABLE 2:** The readers of trypanosomatid histone modifications^*a*^

Domain/module	Modification mark recognized	Organism	No. of readers identified through genome sequence	Specific protein(s) investigated	Any identified property or function related to recognition of modified histone (reference)
Bromodomain	Acetyllysine	T. brucei	6	TbBDF1 to 5	TbBDF2 binds acetylated N-terminal tail of H2AZ (92). TbBDF3 colocalizes with H4K10Ac at TSSs (58).
		T. cruzi	5	TcBDF1, TcBDF2, TcBDF3	TcBDF2 is nuclear. Binds to H4acetyK10. Colocalizes with H4K10Ac and H4K14Ac (93).
		*Leishmania* species	5		
Chromo domain	Methyllysine	T. brucei	1		
		T. cruzi	1		
		*Leishmania* species	1		
Tudor domain	Methyllysine	T. brucei	1	TbEsa1/HAT1	Structure of its N-terminal Tudor domain solved. No methylhistone binding activity detectable (94).
		T. cruzi	1		
		*Leishmania* species	1		
PWWP	Methyllysine	T. brucei	3	TbTFIIS2-1	No methylhistone binding activity detectable in TbTFIIS2-1.
				TbTFIIS2-2	TbTFIIS2-2 binds H4K17me3 and H3K32me3 (96).
		T. cruzi	3		
		*Leishmania* species	3		
PHD finger	Methyllysine	T. brucei	4		
		T. cruzi	4		
		*Leishmania* species	4		

a Note that the large numbers of WD40 and ankyrin repeat-containing proteins that have been annotated but not characterized are not included in the table.

The only known module that recognizes and binds to acetyllysine residues, the bromodomain carries a left-handed four-helix bundle connected by two loops that form the hydrophobic pocket into which the acetyllysine residue docks. Bromodomains have been located in chromatin remodeling factors and transcriptional coactivators. In budding yeast, the acetylation of H4 tails promotes deposition of H2A.Z into nucleosomes by the bromodomain-containing SWR1 complex (98). While a four-protein ISWI complex has been characterized in T. brucei and a multiprotein ISWI complex has been identified in T. cruzi also, none of the components carry an apparent bromodomain (99, 100). Of the six T. brucei bromodomain factors (BDFs), TbBDF3 was the only one to colocalize with H4acetylK10, though direct binding to H4acetylK10 was not demonstrated (63). More recently, H2A.Z and H2B.V were identified as interacting partners of TbBDF2 using a proteomics approach, suggesting the possibility of TbBDF2 binding acetylated H2A.Z (101). In contrast, the nuclear T. cruzi BDF2 colocalized with H4acetylK10 and H4acetylK14 but not with H4acetylK4, also immunoprecipitating with H4acetylK10 (102)—underscoring the fact that the BDF2 orthologs may not be functionally conserved between these closely related organisms. Any direct role these BDFs may play in transcriptional events remains to be determined.

**FIG 2:**
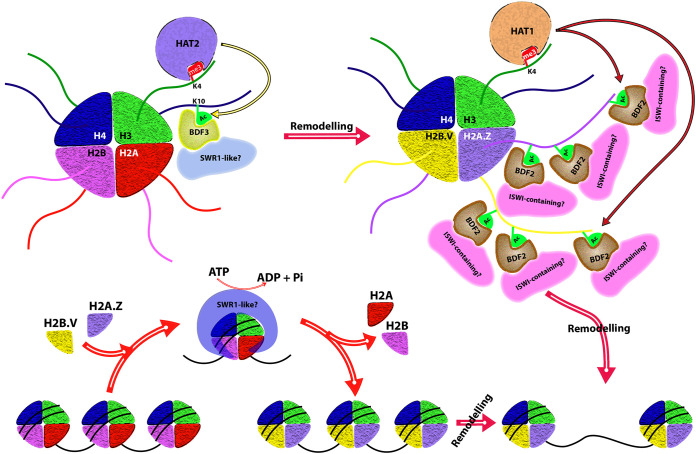
Possible cross talk between histone modifications at the TSSs may govern transcriptional activation. A hypothesized sequence of events follows. Methylation of H3K4 at or very near TSSs within the dSSRs by a hitherto unidentified SET domain methyltransferase is followed by binding of TbHAT2 to H3K4me3 via its chromodomain, suitably positioning it for mediating H4K10 acetylation. TbBDF3 as part of a larger hitherto unidentified SWR1-like chromatin remodeling complex is recruited to H4K10Ac, and the ATP-dependent chromatin remodelers in the complex now arbitrate H2A.Z and H2B.V deposition in these nucleosomes. TbHAT1 is tethered to H3K4me3 via its Tudor domain, positioning it appropriately for hyperacetylating H2A.Z and H2B.V at their N-terminal tails. TbBDF2 associates with acetylated H2A.Z as part of a larger complex of proteins (a larger ISWI complex than the already characterized TbISWI?), and chromatin remodelers inherent in this complex now facilitate the unraveling of nucleosomal structure at the TSSs, resulting in the activation of transcription.

Readers of methylhistones may carry chromodomains, PHD fingers, Tudor domains, and PWWP domains. The T. brucei HAT2 carries a weak chromodomain (this domain has a three-stranded anti-parallel β sheet at the N-terminal end, folding against a C-terminal α-helix) and thus may bind to a methylated histone (63). As TbHAT2 “writes” the H4acetylK10 modification, which localizes with H3K4me3 marks at dSSRs, the authors propose that H4K10 acetylation may be preceded by the interaction of the chromodomain of HAT2 with the H3K4me3 residue. A Tudor domain (a 60-amino-acid-long domain with a conserved antiparallel multistranded β barrel fold) was identified in TbHAT1 and its structure was recently solved, though no methylhistone-binding activity was discernible *in vitro* (103). TbHAT1 may also bind to H3K4me3 at the TSSs via its Tudor domain and thereby be anchored for carrying out acetylation of H2A.Z. In the light of data from various groups (18, 63, 99, 101, 103, 104), it is tempting to speculate on a possible sequence of events at the dSSRs involving cross talk between the different histone PTMs and culminating in transcriptional activation (Fig. 2), though further detailed investigations are needed to support the proposed hypothesis. Two of the three TFIIS orthologs in T. brucei (TFIIS2-1 and TFIIS2-2) carry a PWWP domain; however, only one of them—TFIIS2-2—binds methyl-histones, at H4K17me3 and H3K32me3 (105). If these two marks are indeed synonymous with the universal H4K20me3 and H3K36me3 marks, the complex nature of the downstream effects of histone PTMs becomes apparent when considering the fact that while both these universal marks have been associated with transcriptional repression, the latter has also been associated with transcriptional activation.

## ROLE OF 3D GENOME ARCHITECTURE AND SPATIAL ORGANIZATION WITHIN THE NUCLEUS IN REGULATING DISEASE PATHOGENESIS

The dynamics of the spatial organization of chromatin in the nucleus have an impact on gene expression and regulation. Chromatin conformation capture experiments have allowed the investigation of these aspects, including the distribution of transcriptionally active regions in the three-dimensional (3D) architecture of the genome. The power of the more recently developed Hi-C–Seq technique has been exploited to carry out in-depth analyses of intra- and interchromosomal interactions, allowing us a snapshot of which chromosomes are grouped together in the same territory within the nucleus as well as which regions of different chromosomes are clustered together. This method uses formaldehyde to cross-link nuclear DNA *in vivo*, followed by the digestion of the cross-linked DNA with a restriction enzyme and the labeling of the ends so generated with a biotinylated nucleotide before ligating the fragments together. The biotinylated fragments are collected using streptavidin beads and subjected to next-generation sequencing.

Monoallelic expression of VSGs in T. brucei coupled to the periodic switching of the expressed VSG is the primary mechanism by which this parasite evades the host immune system. By overriding allelic exclusion to generate parasites expressing a mosaic VSG coat, Aresta-Branco et al. recently found that such parasites show very poor survival within the animal host (106). The subtelomeric VSG gene arrays in T. brucei are located at the nuclear periphery, and analysis using the Hi-C technique demonstrated that the chromatin at these subtelomeric arrays was highly compact compared to other genomic regions, linking this repressive environment to VSG gene silencing (29). The active ES is located in an extranucleolar PolI transcription factory—a structure named expression site body (ESB). During transcriptional VSG switching, the ES in the ESB is replaced with another ES, which becomes the site of the new active VSG gene. Muller et al. (29) demonstrated the role of histone variants H3.V and H4.V in connecting the local chromatin environment with genome spatial organization and antigenic variation. Their Hi-C and ATAC-Seq studies revealed that the concurrent loss of these two histone variants led to the repositioning of the silent ESs as well as increased accessibility of DNA along the entire lengths of the silent ESs, favoring VSG switching by recombination events between the now closely positioned ESs. The loss of H3.V and H4.V was also accompanied by the simultaneous expression of more than one VSG (usually two) in some cells. Thus, the two histone variants appear to be playing a role in engineering the 3D organization of the genome, which in turn is an important element in the control of antigenic variation and disease progression.

The use of antigenic variation by protozoan parasites for surmounting the host’s defense systems is not unique to T. brucei, with Plasmodium falciparum also using this strategy. The parasite harbors 60 *var* genes encoding variants of the erythrocyte membrane protein 1 (EMP1), a highly antigenic adhesin, only one of which is expressed on the surface of the infected red blood cell (iRBC) at a given time. The silent *var* genes are grouped together in heterochromatinized regions at the nuclear periphery that are marked by H3K9me3 and coated with HP1, the hallmark of repressed chromatin. A study by Jiang et al. (107) revealed the expression of the *var* genes to be controlled by a histone methyltransferase P. falciparum SETvs (PfSETvs) that mediates H3K36 trimethylation at the promoters and through the bodies of the *var* genes, leading to repression. Parasites devoid of HP1 or PfSET2 simultaneously express almost all *var* genes on the iRBC surface, making them easy targets of the host immune system and leading to parasite clearance (107, 108). PfSIR2A and PfSIR2B also help maintain silencing of *var* genes. The H3K32 methylation mark in trypanosomatids may be synonymous to the H3K36 methylation event of other eukaryotes. It remains to be seen whether a similar mechanism is part of the strategies used by T. brucei to modulate the expression of the VSG genes. The single active *var* gene in P. falciparum is placed in a different (euchromatic) environment that is marked by H3K4me3 and acetylation at H3K9/H3K27/H4K8 (109). Using Hi-C experiments to analyze the 3D genome organization, Bunnik et al. (110) found that the virulence genes were clustered together, allowing for higher frequency of recombination events between the *var* genes and thus increasing the repertoire of antigens that may be expressed. It would be interesting to determine the distribution pattern of the virulence genes of T. cruzi and *Leishmania* species to see whether the virulence genes are clustered in the 3D architecture of their genomes. The virulence genes of *Toxoplasma* are not (110), suggesting that spatial clustering of virulence genes may be a feature that has evolved in pathogens where recombination between virulence genes of multigene families gives the organism a distinctive survival advantage within the host.

## CONCLUDING REMARKS AND FUTURE PERSPECTIVES

The fledgling area of trypanosomatid epigenetics research has expanded rapidly over the past decade or so. An extensive assortment of histone PTMs have now been identified; however, the functional significance of only a handful of them is known at present. Lacunae in our knowledge of histone methylation events are particularly apparent. This is largely due to the presence of so many putative lysine methyltransferases in these organisms. An aggressive approach toward determining the target sites as well as *in vivo* roles of these SET domain proteins would involve creating knockout/knockdown mutants and using high-resolution mass spectrometry to examine their histone PTMs, in addition to analyzing their phenotypes. Alternatively, a battery of modification-specific antibodies would have to be raised to analyze specific methylation events in mutant cells. Recent advances in the use of CRISPR-Cas9 technology are expected to accelerate these studies (111). While studies of *Trypanosoma* are forging ahead, information regarding the global landscape of histone PTMs in *Leishmania* would give a fresh impetus to studies in these organisms as well. A largely unexplored area is the identification and characterization of complex chromatin machinery. Several large multiprotein complexes possessing readers and chromatin remodelers (SWR1 complex), writers and erasers (SAGA complex), or writers and chromatin remodelers (NuA4 complex) have been identified in yeast. Multiprotein epigenetic complexes of similar complex nature have also been identified in *Plasmodium* and *Toxoplasma* species (112, 113). However, very little information is available with respect to trypanosomatids, with the T. brucei ISWI (TbISWI) and T. cruzi ISWI (TcISWI) complexes being the only multiprotein complexes reported so far. TbISWI has been found to be enriched at PolII TSSs in addition to playing a role in VSG expression site regulation (99, 104). The identification of proteins associated with the writers and readers will draw the field into new territories. Several studies, particularly in cancer cells, have targeted histone writers and erasers as sites for therapeutic intervention. The enzymes that write/erase histone modifications in trypanosomatids may serve as potential drug targets. These aspects need further investigations.
