# Erratum for Saha, “Histone Modifications and Other Facets of Epigenetic Regulation in Trypanosomatids: Leaving Their Mark”

**DOI:** 10.1128/mBio.02247-21

**Published:** 2021-08-31

**Authors:** Swati Saha

**Affiliations:** a Department of Microbiology, University of Delhi South Campus, New Delhi, India

## ERRATUM

Volume 11, no. 5, https://doi.org/10.1128/mBio.01079-20. During manuscript revision, the original reference numbers in Tables 1 and 2 were erroneously retained. The revised Tables 1 and 2 (below) have the correctly numbered references.

**TABLE 1:** The writers and erasers of trypanosomatid histone modifications

Class of modifier	Type	Organism	No. of modifiers identified through genome sequence	Protein	Histone substrate if identified	Functional role (s) if known (reference)^*a*^
Histone acetyltransferase	MYST family	T. brucei	3	HAT1	H2A.Z	Essential. Nuclear. Replication, transcription (40, 18).
				HAT2	H4K10	Essential. Nuclear. Transcription (40, 18).
				HAT3	H4K4	Nonessential. Nuclear. DNA repair (41, 97).
		T. cruzi	4	HAT1		
				HAT2		
				HAT3		
				HAT4		
		*Leishmania* species	4	HAT1		
				HAT2	H4K10	Essential. Nuclear. Transcription (22).
				HAT3	H4K4	Nonessential. Nuclear. DNA repair (23).
				HAT4	H4K14 *in vitro*	Nonessential. Cytosolic except at G2M. Cell cycle control (42, 43).
	GNAT family	T. brucei	2	Elp3a		Nonessential. Nuclear. No role determined yet (44).
				Elp3b		Nonessential. Nuclear. rDNA transcription (44).
		T. cruzi	2	Elp3a		
				Elp3b		
		*Leishmania* species	2	Elp3a		
				Elp3b		
Histone deacetylase	Zn^2+^-dependent HDACs	T. brucei	4	DAC1		Essential. Nuclear. Telomeric silencing antagonist (27, 28).
				DAC2		Nonessential. Cytoplasmic (27).
				DAC3		Essential. Nuclear. Silencing of ESs of VSGs (27, 28).
				DAC4		Nonessential. Cytoplasmic. Cell cycle progression (27).
		T. cruzi	4	DAC1		
				DAC2		
				DAC3		
				DAC4		
		*Leishmania* species	3	DAC1		
				DAC3		
				DAC4		
	NAD^+^-dependent sirtuins	T. brucei	3	Sir2rp1	H2A, H2B *in vitro*	Nonessential. Nuclear. DNA repair. Subtelomeric transcription control (31, 32, 33).
				Sir2rp2		Nonessential. Mitochondrial.
				Sir2rp3		Nonessential. Mitochondrial.
		T. cruzi	2	Sir2rp1		Cytoplasmic.
				Sir2rp3		Mitochondrial.
		*Leishmania* species	3	Sir2rp1		Essential. Cytoplasmic. α-Tubulin deacetylation.
				Sir2rp2		Mitochondrial.
				Sir2rp3		Mitochondrial.
Histone methyltransferase	SET domain-containing	T. brucei	∼25−30			
		T. cruzi	∼25−30			
		*Leishmania* species	∼25−30			
	DOT1-related	T. brucei	3	DOT1A	H3K76me2	Essential. Replication (25, 46).
				DOT1B	H3K76me3	Nonessential. VSG silencing and transcriptional switching, cell cycle control (46, 73).
				DOT1 putative		
		T. cruzi	3	DOT1A		
				DOT1B	H3K76me3	Nonessential. Promotes mitosis and cytokinesis (48).
				DOT1 putative		
		*Leishmania* species	3	DOT1A		
				DOT1B		
				DOT1 putative		
	PRMT	T. brucei	5	PRMT1		Nonessential. Regulation of cell’s response to starvation.
				PRMT3		
				PRMT5		Nonessential. Cytoplasmic.
				PRMT6	H3 and H4 *in vitro*	Nonessential. Regulation of cytokinesis. Interacts with core histones (51).
				PRMT7	H4 peptides *in vitro*	Nonessential. Cytoplasmic.
		T. cruzi		PRMT1		
				PRMT3		
				PRMT5		
				PRMT6		
				PRMT7		
		*Leishmania* species	5	PRMT1		
				PRMT3		
				PRMT5		
				PRMT6		
				PRMT7		Nonessential. Cytosolic. RNA biology (50).
Histone demethylases	Jumonji family	T. brucei	4			
		T. cruzi	4			
		*Leishmania* species	4			

a References are cited here only where related to histones and/or DNA-related processes.

**TABLE 2:** The readers of trypanosomatid histone modifications^*a*^

Domain/module	Modification mark recognized	Organism	No. of readers identified through genome sequence	Specific protein(s) investigated	Any identified property or function related to recognition of modified histone (reference)
Bromodomain	Acetyllysine	T. brucei	6	TbBDF1 to -5	TbBDF2 binds acetylated N-terminal tail of H2AZ (101). TbBDF3 colocalizes with H4K10Ac at TSSs (63).
		T. cruzi	5	TcBDF1, TcBDF2, TcBDF3	TcBDF2 is nuclear. Binds to H4acetyK10. Colocalizes with H4K10Ac and H4K14Ac (102).
		*Leishmania* species	5		
Chromo domain	Methyllysine	T. brucei	1		
		T. cruzi	1		
		*Leishmania* species	1		
Tudor domain	Methyllysine	T. brucei	1	TbEsa1/HAT1	Structure of its N-terminal Tudor domain solved. No methylhistone binding activity detectable (103).
		T. cruzi	1		
		*Leishmania* species	1		
PWWP	Methyllysine	T. brucei	3	TbTFIIS2-1	No methylhistone binding activity detectable in TbTFIIS2-1.
				TbTFIIS2-2	TbTFIIS2-2 binds H4K17me3 and H3K32me3 (105).
		T. cruzi	3		
		*Leishmania *species	3		
PHD finger	Methyllysine	T. brucei	4		
		T. cruzi	4		
		*Leishmania * species	4		

a Note that the large numbers of WD40 and ankyrin repeat-containing proteins that have been annotated but not characterized are not included in the table.

